# Cerebral small-vessel disease at high altitude: A comparison of patients from plateau and plain

**DOI:** 10.3389/fneur.2023.1086476

**Published:** 2023-03-09

**Authors:** Junlong Shu, Wen Fei, Jing Zhang, Fan Li, Yu Hao, Zhijie Ding, Tseyang, Drolma, Shiyong Ji, Weiwei Zhao, Yaxiong Hu, Wei Sun, Yining Huang, Yuhua Zhao, Wei Zhang

**Affiliations:** ^1^Department of Neurology, Peking University First Hospital, Beijing, China; ^2^Beijing Key Laboratory of Neurovascular Disease Discovery, Beijing, China; ^3^Department of Neurology, People's Hospital of Tibet Autonomous Region, Lhasa, China

**Keywords:** cerebral small-vessel disease, white matter hyperintensity, plateau, plain, stroke, lacune of presumed vascular origin

## Abstract

**Background and purpose:**

Cerebral small-vessel disease (CSVD) is prevalent worldwide and one of the major causes of stroke and dementia. For patients with CSVD at high altitude, a special environmental status, limited information is known about their clinical phenotype and specific neuroimaging change. We investigated the clinical and neuroimaging features of patients residing at high altitude by comparing with those in the plain, trying to explore the impact of high altitude environment on CSVD.

**Methods:**

Two cohorts of CSVD patients from the Tibet Autonomous Region and Beijing were recruited retrospectively. In addition to the collection of clinical diagnoses, demographic information and traditional vascular risk factors, the presence, location, and severity of lacunes and white matter hyperintensities were assessed by manual counting and using age-related white matter changes (ARWMC) rating scale. Differences between the two groups and influence of long-term residing in the plateau were analyzed.

**Results:**

A total of 169 patients in Tibet (high altitude) and 310 patients in Beijing (low altitude) were enrolled. Fewer patients in high altitude group were found with acute cerebrovascular events and concomitant traditional vascular risk factors. The median (quartiles) ARWMC score was 10 (4, 15) in high altitude group and 6 (3, 12) in low altitude group. Less lacunes were detected in high altitude group [0 (0, 4)] than in low altitude group [2 (0, 5)]. In both groups, most lesions located in the subcortical (especially frontal) and basal ganglia regions. Logistic regressions showed that age, hypertension, family history of stroke, and plateau resident were independently associated with severe white matter hyperintensities, while plateau resident was negatively correlated with lacunes.

**Conclusion:**

Patients of CSVD residing at high altitude showed more severe WMH but less acute cerebrovascular events and lacunes in neuroimaging, comparing to patients residing at low altitude. Our findings suggest potential biphasic effect of high altitude on the occurrence and progression of CSVD.

## Introduction

Cerebral small-vessel disease (CSVD) is one of the most prevalent syndromes worldwide which was thought contributing to ~25% of strokes and 45% of dementia cases (1). Various pathophysiological changes such as hypoxic-ischemic injury (2, 3), breakdown of the blood–brain barrier (4), loss of autoregulation (2), activation of the innate immune system (3), and protein elimination failure (5) have been found to be involved in the development of disease, which lead to landmark neuroimaging changes such as white matter hyperintensity (WMH), lacune of presumed vascular origin and microbleed. Age and hypertension are the most recognized risk factors of CSVD. Other traditional vascular risk factors, such as cigarette smoking, diabetes mellitus, dyslipidemia, obstructive sleep apnea, and chronic kidney disease, have been fully studied although the conclusions were controversial (1). Environmental factors such as the hypobaric hypoxia at high altitude have been reported to have an effect on CSVD (6, 7), but it has been rarely investigated.

It is estimated that over 500 million humans (6.58% of the total population) live above 1,500 m (8). As altitude increases, the amount of gas molecules in the air decreases, resulting in a drop in barometric pressure and partial pressure of oxygen. People who ascend to high altitude and exposed to hypobaric hypoxia will suffer a significant decrease of arterial and tissue partial pressure of oxygen in the brain (9). A series of physiological responses will be triggered. In acute phase, cerebral blood flow increases firstly and an increase in vascular permeability may occur, due to hypoxia-induced endothelial dysfunction, which could lead to headache and even cerebral edema in unacclimatised individuals (10, 11). During chronic hypoxia, cerebral blood flow returns toward the baseline level gradually, and oxygen delivery to brain will be compensated by other adaptation including an increase in hemoglobin concentration, hematocrit, red blood cell count and vascular density, resulting in an increase in tissue partial pressure of oxygen of the brain at an equal atmospheric partial pressure of oxygen, as suggested by animal experiments (11). The changed hematological and hemodynamic state after chronic exposure to hypoxia has been considered to play a role in the occurrence of CSVD, especially on WMH and lacunar infarction, which are closely related to endothelial dysfunction and oxygen supply (3, 4). However, the limited research data mainly focus on the association between high altitude exposure and the occurrence, subtype or outcome of stroke (12–16), while the other changes due to small vessel impairment are rarely involved (17). To explore the impact of chronic high altitude exposure on CSVD, we conduct the study comparing clinical and neuroimaging difference of CSVD between patients from the plain and the plateau.

## Methods

### Study design and population

Two cohorts of CSVD patients were retrospectively recruited from Peking University First Hospital (altitude between 0–50 m) and the People's Hospital of Tibet Autonomous Region (altitude between 3,000–5,000 m) in China. We searched the medical records in the database of the Inpatient Department of Neurology and selected eligible patients as our study population. Patients in Beijing were recruited between 2013–2019 from the neurology ward of Peking University First Hospital, while patients in Tibet were recruited between 2017–2019 from the neurology ward of the People's Hospital of Tibet Autonomous Region. All studies were performed with approval from the Ethics Committee of Peking University (IRB00001052-17018) and the Tibet Autonomous Region (ME-TBHP-19-37). Informed consents were obtained from all patients.

### Inclusion criteria

According to the International Classification of Diseases 10th Revision, patients diagnosed with cerebrovascular disease (code I61–I69) were included if the following criteria were met.

First, the patients have received head magnetic resonance imaging (MRI) examination and the images of MRI showed more than 1 characteristic neuroimaging changes of CSVD, including recent small subcortical infarcts, lacunes of presumed vascular origin, WMH of presumed vascular origin, enlarged perivascular space, and cerebral microbleeds, following the definition of STandards for Reporting Vascular changes on neuroimaging (STRIVE) (18).

Second, the patients have not been found with noteworthy stenosis of large vessels (defined as a stenosis more than 50%) or vascular malformation after adequate vascular assessments (including carotid ultrasound or computed tomography angiography for the extracranial arteries and transcranial Doppler sonography, magnetic resonance angiography, or computed tomography angiography for the intracranial arteries).

Third, the patients have resided locally for more than 10 years.

### Exclusion criteria

Patients who lacked information of head MRI or vascular assessments, or complicated by other diseases including atrial fibrillation, hemopathy, inflammatory demyelinating disease, tumor, or leukodystrophy which can cause similar neuroimaging changes other than CSVD, were excluded. Besides, patients who resided in Beijing for more than 10 years but had a history of tourism or residence in plateau were also excluded.

### Clinical data collection

Clinical diagnosis, demographic information, and common vascular risk factors, including hypertension, diabetes, dyslipidemia, coronary heart disease, and smoking, were collected from medical records. The clinical diagnoses were categorized into four clinical conditions when summarized: transient ischemic attack (TIA) or ischemic stroke, cerebral hemorrhage, vascular cognitive impairment or vascular parkinsonism, and CSVD with non-specific symptoms (such as dizziness, headache, numbness, depression, insomnia, etc.). Serum homocysteine concentrations were documented and were defined as hyperhomocysteinemia when more than 15 μmol/L. A family history of stroke was identified as positive when either of the patient's parents had suffered from hemorrhage or ischemic stroke.

### Neuroimaging evaluation

All patients included have received a head MRI scan. Basic scanning sequences included T1-weighted imaging, T2-weighted fluid-attenuated inversion recovery imaging, and diffusion weighted imaging. It is noteworthy that about half of the patients in Beijing lack T2-weighted images because of setting of the scan protocol. Besides, gradient-recalled echo or susceptibility-weighted imaging was performed in most patients in Beijing, but only in a few patients in Tibet. MRI data in Beijing were acquired on two scanners (SIGNA EXCITE 1.5T; General Electric Medical Systems, Milwaukee, WI, USA; Achieva 3.0T; Philips Medical Systems, Netherlands) with a slice thickness of 6.0 mm. MRI data in Tibet were acquired on a 3.0T scanner (Magnetom Verio; Siemens Healthcare, Erlangen, Germany) with a slice thickness of 6.0mm.

Two independent raters (JZ, WF), blinded to the patients' clinical information or outcomes, evaluated the MRI data. Due to the difference in the scanning sequence of the head MR images provided by the two hospitals, only lacunes and WMH were evaluated. Lacunes were detected according to the definition in the consensus of STRIVE (18), with the different anatomical regions documented and classified into frontal, parieto-occipital, temporal, basal ganglia, brainstem, and cerebellum. The age-related white matter changes (ARWMC) rating scale (19) was used to evaluate the presence and severity of WMH, in which the white matter lesions were scored 0, 1, 2, and 3 in different brain regions, including the frontal, parieto-occipital, temporal, infratentorial/cerebellum, and basal ganglia regions, and a sum of all regional scores called ARWMC Score (ranges from 0 to 30) was adopted to show the load of WMH in the whole brain. Evaluations were performed in the picture archiving and communication system of each hospital. Unified training was initially conducted *via* video conference by a senior neurologist (JLS) with more than 5 years' experience in neuroimaging. Consensus was achieved after training and before formal evaluation. The intraclass correlation coefficient of intra-rater reliability was 0.95 [95% confidence interval (CI), 0.92–0.98] and the inter-rater reliability was 0.90 (95% CI, 0.86–0.92).

### Statistical analysis

Statistical analyses were performed using statistical software (Statistical Package for Social Sciences, version 24.0; IBM SPSS Statistics, Armonk, NY, USA). Data are presented as frequency, mean ± standard deviation, or median (quartiles), depending on the nature of the data. The χ^2^ test and the Fisher's exact test were used to test for differences in the listed risk factors and the presence and anatomic distribution of lacunes or WMH between the two groups. The student's *t*-test was used to compare age, while the Mann–Whitney *U*-test was used to test the disparity of the ARWMC score and the lacune count, between the two groups. Binary logistic regression was performed to determine the impact of various risk factors on severe WMH and occurrence of lacunes (including age, sex, hypertension, diabetes, coronary heart disease, cigarette smoking, hyperhomocysteinemia, dyslipidemia, family history of stroke, and residing in the plateau) using the forward stepwise regression method based on the maximum likelihood estimation to screen the independent variables. For all analyses, *p* < 0.05 was considered statistically significant.

## Results

### Clinical and neuroimaging information of study population

**Figure 1:**
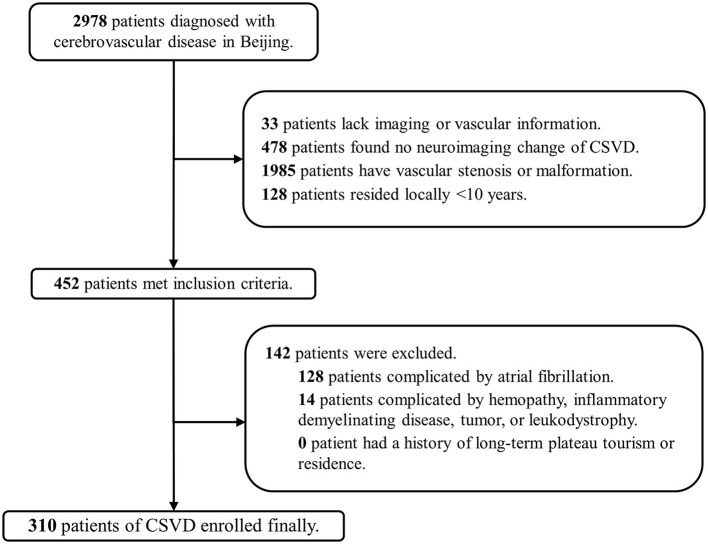
Flowchart of patient recruitment in Beijing. CSVD, cerebral small-vessel disease.

**Figure 2:**
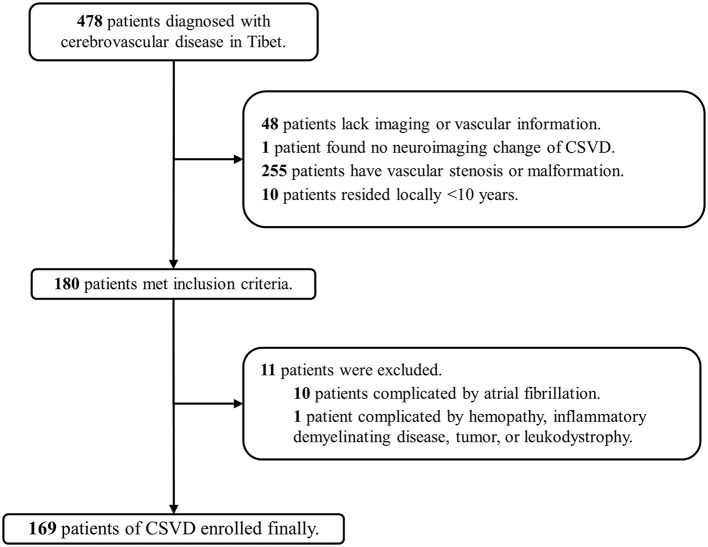
Flowchart of patient recruitment in Tibet. CSVD, cerebral small-vessel disease.

A total of 2,978 patients in Beijing and 478 patients in Tibet who were diagnosed with cerebrovascular disease were included in our screening. Finally, 310 patients (10.4%) in Beijing (low altitude group) and 169 patients (35.4%) in Tibet (high altitude group) were enrolled in the two cohorts (Figures 1, 2). The average age was 63 ± 12 years old, and 300 (62.6%) were male. The proportion of traditional vascular risk factors are shown in Table 1. A total of 457 (95.4%) patients were found with WMH, and 303 (63.3%) had been detected to have lacunes. The median ARWMC score was 7 (interquartile range, 4–13), while the median count of lacunes was 2 (interquartile range, 0–4).

**Table 1:** Clinical and neuroimaging information of study population.

		**All (*n* = 479)**	**Low altitude (*n* = 310)**	**High altitude (*n* = 169)**	***p*-value**
Clinical condition	TIA/ischemic stroke (%)	219 (45.7)	202 (65.2)	17 (10.1)	< 0.001
	Cerebral hemorrhage (%)	36 (7.5)	36 (11.6)	0	
	VCI or VP (%)	13 (2.7)	4 (1.3)	9 (5.3)	
	CSVD found with non-specific symptoms (%)	211 (44.1)	68 (21.9)	143 (84.6)	
Age, mean ± SD		63 ± 12	63.1 ± 11.7	62.1 ± 13.9	0.389
Male, *n* (%)		300 (62.6)	209 (67.4)	91 (53.8)	0.003
Hypertension, *n* (%)		346 (72.2)	258 (83.2)	88 (52.1)	< 0.001
Diabetes, *n* (%)		114 (23.8)	93 (30)	21 (12.4)	< 0.001
Coronary heart disease, *n* (%)		58 (12.1)	45 (14.5)	13 (7.7)	0.029
Dyslipidemia, *n* (%)		214 (44.7)	184 (59.4)	30 (17.8)	< 0.001
Hyperhomocysteinemia, *n* (%)		129 (27.5)	100 (32.3)	29 (18.2)	0.001
Cigarette, *n* (%)		179 (37.4)	137 (44.2)	42 (24.9)	< 0.001
Family history of stroke, *n* (%)		103 (21.5)	100 (32.3)	3 (1.8%)	< 0.001
White matter hyperintensity, *n* (%)		457 (95.4)	292 (94.2)	165 (97.6)	0.086
ARWMC score, median (quartiles)		7 (4, 13)	6 (3, 12)	10 (4, 15)	< 0.001
Presence of lacune, *n* (%)		303 (63.3)	225 (72.6)	78 (46.2)	< 0.001
Lacune count, median (quartiles)		2 (0, 4)	2 (0, 5)	0 (0, 4)	< 0.001

TIA, transient ischemic attack; VCI, vascular cognitive impairment; VP, vascular parkinsonism; CSVD, cerebral small-vessel disease; SD, standard deviation; ARWMC, age related white matter change.

**Figure 3:**
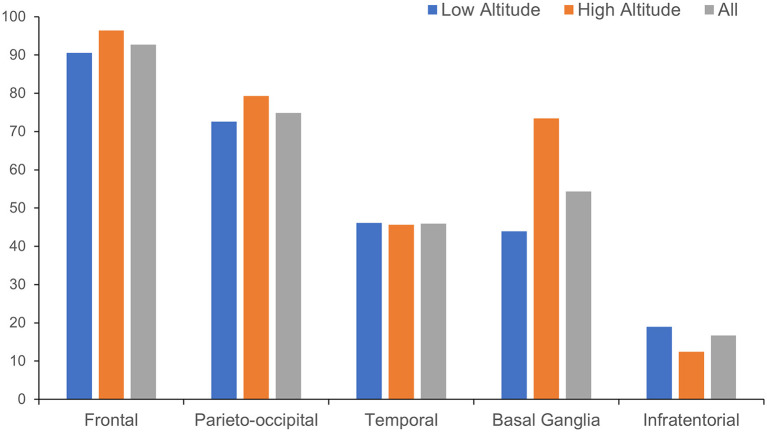
Distribution of white matter hyperintensity in different anatomical regions.

**Figure 4:**
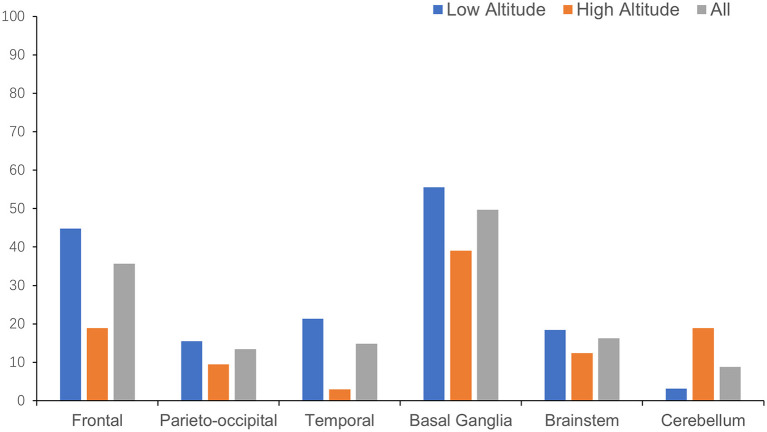
Distribution of lacune in different anatomical regions.

The anatomical distributions of different neuroimaging change are shown in Table 2. WMH tended to appear in subcortical areas, especially the frontal and parieto-occipital regions, and was found in the basal ganglia in more than half of the patients (Figure 3). Lacunes were most often found in the basal ganglia, followed by the frontal lobe (Figure 4).

**Table 2:** Distribution of white matter hyperintensity and lacune in study population.

	**All (*n* = 479)**	**Low altitude (*n* = 310)**	**High altitude (*n* = 169)**	***p*-value**
**White matter hyperintensity**				
Subcortical (frontal), *n* (%)	444 (92.7)	281 (90.6)	163 (96.4)	0.020
Subcortical (parieto-occipital), *n* (%)	359 (74.9)	225 (72.6)	134 (79.3)	0.105
Subcortical (temporal), *n* (%)	220 (45.9)	143 (46.1)	77 (45.6)	0.905
Basal ganglia, *n* (%)	260 (54.3)	136 (43.9)	124 (73.4)	< 0.001
Infratentorial/cerebellum, *n* (%)	80 (16.7)	59 (19.0)	21 (12.4)	0.064
**Presence of lacune**				
Frontal lobe, *n* (%)	171 (35.7)	139 (44.8)	32 (18.9)	< 0.001
Parieto-occipital lobe, *n* (%)	64 (13.4)	48 (15.5)	16 (9.5)	0.064
Temporal lobe, *n* (%)	71 (14.8)	66 (21.3)	5 (3.0)	< 0.001
Basal ganglia, *n* (%)	238 (49.7)	172 (55.5)	66 (39.1)	0.001
Brainstem, *n* (%)	78 (16.3)	57 (18.4)	21 (12.4)	0.091
Cerebellum, *n* (%)	42 (8.8)	10 (3.2)	32 (18.9)	< 0.001

### Comparison of high and low altitude groups

**Figure 5:**
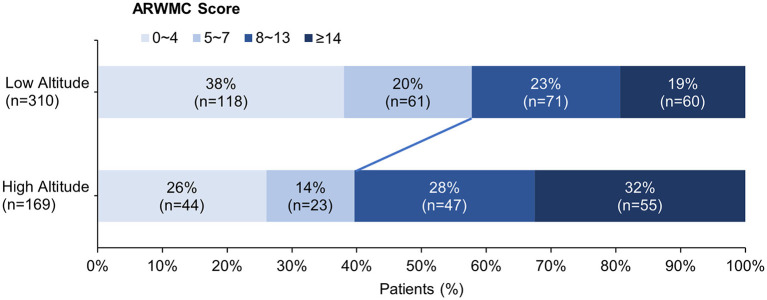
Ordinal distribution of age-related white matter change (ARWMC) Scores for cerebral small-vessel disease patients. The severity of white matter hyperintensity was evaluated using ARWMC rating scale. The distribution of scores in each group is shown by accumulating bar charts. There is a significant shift to left in the plateau group, suggest patients in the plateau group tend to have more severe white matter hyperintensity.

**Figure 6:**
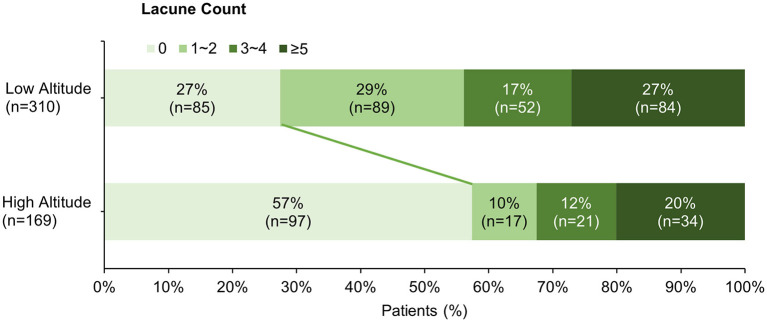
Ordinal distribution of Lacune count for cerebral small-vessel disease patients. The distribution of lacune count in each group is shown by accumulating bar charts. There is a significant shift to right in the plateau group, suggest patients in the plateau group tend to have less lacunes compared with patients in the plain group.

The clinical and neuroimaging characters of two groups are also shown in Table 1. More than half of the patients in low altitude group were diagnosed with TIA or ischemic stroke (65.2%), followed by CSVD found with non-specific symptoms (21.9%), while 143 of the 169 patients (84.6%) in high altitude group were CSVD found with non-specific symptoms and only 10.1% of the patients had TIA or stroke. The low altitude group had a higher proportion of male patients (67.4 vs. 53.8%, *p* = 0.003), and also a higher proportion of traditional vascular risk factors, such as hypertension (83.2 vs. 52.1%, *p* < 0.001), diabetes (30 vs. 12.4%, *p* < 0.001), cigarette (44.2 vs. 24.9%, *p* < 0.001), etc. In neuroimaging, patients in high altitude group had more severe WMH than patients in low altitude group [ARWMC score 10 (4, 15) vs. 6 (3, 12); *p* < 0.001], although nearly all patients in both groups were found to have WMH (97.6 vs. 94.2%, *p* = 0.086). On the contrary, the detection of lacune in patients of high altitude group was fewer than in patients of low altitude groups (46.2 vs. 72.6%), with significant difference (*p* < 0.001). Detailed ordinal distributions of ARWMC score and lacune count in the two groups are shown in Figures 5, 6, showing the tendency of more severe WMH and less lacunes in high altitude group.

For the anatomical distribution, patients in high altitude group shared a similar mode with patients in low altitude group in distribution of WMH and lacune. Subcortical area and basal ganglia were the main susceptible areas of WMH and lacune. Comparing with patients in low altitude group, patients in high altitude group were more likely to have WMH in basal ganglia (73.4 vs. 43.9%, *p* < 0.001) and lacune in cerebellum (18.9 vs. 3.2%, *p* < 0.001; Table 2).

### Impact of multiple risk factors on severe WMH and lacune

According to the median ARWMC score of all patients, an ARWMC score >7 was identified as severe WMH. Logistic regression analysis showed that age, hypertension, family history of stroke, and residing in the plateau were independently associated with severe WMH (Table 3). Patients residing in the plateau were four times more likely to suffer from severe WMH compared with patients residing in the plain [odds ratio (OR), 4.083; 95% CI, 2.496–6.680; *p* < 0.001]. Meanwhile, logistic regression analysis using the occurrence of lacune as a dependent variable demonstrated that age, hypertension, cigarette smoking, and hyperhomocysteinemia were independent-related factors, while residing in the plateau was negatively correlated with occurrence of lacune (OR, 0.430; 95% CI, 0.276–0.670; *p* < 0.001; Table 4).

**Table 3:** Logistic regression analysis for risk factors associated with severe WMH (ARWMC Score >7).

**Risk factors**	**B**	**SD**	**OR**	**95%CI**	***p*-value**
Age	0.046	0.009	1.047	1.029–1.066	< 0.001
Hypertension	0.619	0.248	1.857	1.142–3.020	0.013
Family history of stroke	1.050	0.262	2.857	1.711–4.770	< 0.001
Resided in plateau	1.407	0.251	4.083	2.496–6.680	< 0.001

B, beta; SD, standard deviation; OR, odds ratio; CI, confidence interval.

**Table 4:** Logistic regression analysis for risk factors associated with lacune.

**Risk factors**	***B***	**SD**	**OR**	**95%CI**	***p*-value**
Age	0.028	0.009	1.028	1.010–1.046	0.002
Hypertension	0.675	0.243	1.964	1.219–3.164	0.006
Cigarette	0.787	0.231	2.196	1.396–3.453	0.001
Hyperhomocysteinemia	0.548	0.253	1.730	1.055–2.838	0.030
Resided in plateau	−0.844	0.226	0.430	0.276–0.670	< 0.001

B, beta; SD, standard deviation; OR, odds ratio; CI, confidence interval.

## Discussion

China encompasses a vast area of high altitude with four plateaus, among which the Qinghai –Tibet plateau is the highest plateau of earth and sometimes called “the roof of the world.” Approximately 80 million people live above 2,500 m in China, among whom nearly 3.7 million living around 4,000 m in Tibet. Previous studies have reported increased cerebral blood flow and blood–brain barrier damage in individuals following ascent to high altitudes, which may result in vasogenic edema and microhemorrhages (20), and indicate a higher risk of cerebrovascular disease. As an important part of cerebrovascular disease, CSVD can not only cause stroke, but also lead to many other chronic symptoms such as cognitive decline and gait disorder, increasing the risk of disability and hospitalization. Viewing from the constitution of cerebrovascular disease in our study, the ratio of CSVD seem to be much higher in high altitude area (35.4 vs. 10.4%) than low altitude area. Many factors could be associated with this disparity, such as the selective admission of inpatients, insufficient in-hospital assessment, restriction of CSVD in inclusion criteria of the study. However, we noted that in the screening, 478 of 2,978 (16.1%) patients with cerebrovascular disease in the low altitude group find no neuroimaging change of CSVD in MRI, while there was only one of 478 patients in the high-altitude group. It indicates the tendency that CSVD might be more prevalent in high altitude area, although no conclusion can be drawn due to the selection bias and further epidemiological investigation is needed. Anyway, the relatively high proportion of CSVD reflects the actual status of inpatients in high altitude area, and more attentions are deserved for this population.

In this study, we chose the inpatients as study population, in which we could obtain more sufficient clinical and neuroimaging information compared with the outpatient and community residents. Strict criteria were used to select the patients of CSVD. We excluded other diseases that may cause similar neuroimaging changes, especially the large vessel stenosis and atrial fibrillation, to reduce the possible confounding in the mechanism or evaluation and get two cohorts of pure CSVD patients relatively. Local residence is necessary to keep the basic difference between the two groups. Since the mobility of patients in Beijing is much higher than that in Tibet, we set the long-term limit of 10 years that was easy for patients to report and excluded the one who had history of tourism or residence in plateau when screening patients for low altitude groups, to ensure the patients enrolled in each group residing at same altitude all the year round.

There are significant differences in the proportions of traditional vascular risk factors between the two groups, especially hypertension, which has been confirmed as the most important risk factor for CSVD other than age (1). Fewer patients were found to have traditional vascular risk factors in high altitude group. Because of the study design, it is arbitrary to conclude that people residing at high altitude have a lower prevalence of traditional risk factors than those residing at low altitude. On the contrary, a systematic review have reported a 2% increase in the prevalence of hypertension in Tibetans with every 100 m increase in altitude (21). Two hypotheses we considered may explain the difference. First, other non-traditional vascular risk factors were introduced, such as the hypobaric hypoxia, low temperature or unique dietary habit at high altitude and ambient particulate matter pollution at low altitude. A part of patients in high altitude group may be affected only by these non-traditional vascular risk factors, which reduced the impact of traditional vascular risk factors on CSVD competitively, resulting in a lower proportion than those in low altitude group. Second, selective bias may exist. It is possible that patient of CSVD who had hypertension or other traditional risk factors in high altitude area were more prone to severe stenosis of large vessels than those in low altitude area, whose data were not collected for our analysis. If it happened, it would lead to a reduction in proportion of traditional vascular risk factors in the final high altitude group. Further research and analysis are needed.

More severe WMH and less lacunes were found in high altitude group. Further logistic regression analysis showed that patients of CSVD residing in plateau had about three times risk increase for suffering from severe WMH and more than 50% risk decrease for lacune after adjusting for potential confounding factors such as age, hypertension, cigarette, compared with those residing in plain. Chronic hypoxia at high altitude seems to have affected CSVD in opposite directions. For one thing, hypoxia may cause and aggravate white matter damage, as suggested in previous research about obstructive sleep apnea (22) and chronic obstructive pulmonary disease (23), which could also keep the patients in a state of hypoxia for a long time. In these conditions, sympathetic activation, altered cerebral blood flow and velocity, and endothelial dysfunction were reported contributing to small-vessel damage and formation of WMH (22–25). However, there was no comparable study about chronic hypoxia patients residing at high altitude before us.

On the other hand, chronic hypoxia seems to be a protective factor of lacune. As a neuroimaging marker of CSVD, lacune marks the healed stage of a small deep brain necrosis (18). The cause of most lacunes is presumed to be small subcortical infarcts or so-called lacunar infarction, although some might be the result of small deep hemorrhages. Fewer patients with lacunes were found in high altitude group in contrast to the more severe WMH. It means that patients of CSVD residing in Tibet might suffer from less lacunar infarctions, or milder even if it happened, which halted the formation of lacune, and indicate a potential protective effect on lacune or lacunar infarction from chronic hypoxia. Selective bias may have a great impact. Most of the patients included in low altitude group had an acute cerebrovascular event, while the most in high altitude group showed only non-specific symptoms. As this is a comparative study based on patients in a single hospital, there is a possibility that some patients with acute ischemic events in Tibet and some patients with only non-specific symptoms in Beijing were not included in the study if they were not admitted to the hospitals we chose, resulting a reduction in the ratio of lacune and stroke in patients at high altitude. However, similar finding was reported in study on subtypes of ischemic stroke at high altitude, with a lower proportion of small-vessel occlusion in patients from Tibet than in those from the plain (3.0 vs. 23.7%; *p* < 0.001) (13). A probable explanation for this phenomenon is the hypoxic acclimation. When humans are continuously exposed to hypoxia, compensatory mechanisms will developed to maintain the oxygen supply, including erythrocytosis, angiogenesis, capillary remodeling and improved ventilatory response (11, 26), which may enhance the brain's resistance to hypoxic-ischemic injury. In studies about stroke and chronic high altitude exposure, Ortiz-Prado E. and colleagues found that prolonged residing at high altitude will reduce the risk of developing stroke and is associated with lower stroke-related mortality (12, 26). This protective effect is stronger if the altitudes range from 2,000 to 3,500 m while residing above 3,500 m may be associated with an increased risk of developing stroke. It is consistent with the high stroke incidence reported in the epidemiological data of Tibet (13), as most of its cities or towns are located above 3,500 m. However, in this study, we found that this protection seems to be effective for lacunar infarction still, even when the patients reside at a very high altitude (Tibet). Angiogenesis or capillary remodeling in the brain may be the key reasons. Because when a small vessel approaches to occlusion, the newborn or remodeling capillary around it can provide a good compensate for its supply area, but it may be invalid when large vessels are occluded due to blood stasis or thrombogenesis caused by the significantly high hematocrit and polycythemia at a very high attitude. More studies about the prevalence of CSVD at different elevations may be helpful.

The anatomical distribution of neuroimaging makers of CSVD is also an important consideration. In both groups, WMHs were mainly distributed in the subcortical regions dominated with frontal white matter, followed by the basal ganglia region, while the lacunes were mainly distributed in the basal ganglia, followed by the frontal cortex and subcortical area, which was more consistent with arteriolosclerosis type of CSVD other than cerebral amyloid angiopathy (27) or cerebral autosomal dominant arteriopathy (28, 29). Patients of CSVD in high altitude group had a higher proportion of WMH in basal ganglia than those in low altitude group, reflecting a more extensive white matter destruction. The relatively sparse distribution of small vessels and the high tissue oxygen demand are proposed to contribute to the distribution pattern of CSVD lesions (3). In patients residing in the plateau, the additional chronic hypoxic conditions make this distribution pattern more prominent, especially WMH in the basal ganglia, which suggests a more severe small vessel involvement (30). Besides, patients residing in plateau were detected to have more lacunes in cerebellum but fewer in temporal region. Poor hypoxic acclimation in cerebrum and angiogenesis around bilateral posterior cerebral arteries might explain this discovery barely, as found in moyamoya disease or syndrome (31). What is more, genetic or ethnic disparities may also make a difference. In the previous study, we found a certain difference in the spatial distribution of lesions between patients of CSVD in China and Germany (32). Yakushi and colleagues also reported a different anatomical distribution of cerebral microbleeds between Eastern and Western populations (33). Further research is needed to verify the correlation and explore detailed mechanism.

## Limitations

Limitations exist in this study. First, as it was a hospital-based comparative study with a limited sample size, our findings may not be generalizable to the whole population that resides in the plateau or the plain. Second, the neuroimaging evaluations of each group were completed separately by two independent raters because of image transmission and privacy protection restrictions, which might have an impact on the reliability of neuroimaging evaluation. Nevertheless, unified training and reliability analysis were performed before the formal evaluation. Third, because of the lack of gradient-recalled echo or susceptibility-weighted sequence imaging in the plateau group, we were unable to assess the impact of high altitude environment on cerebral microbleeds, which is another key neuroimaging marker of CSVD.

## Conclusions

In this comparative study of CSVD patients at different altitudes, we have found that patients residing at high altitude have suffered from less acute cerebrovascular events, owned a lower proportion of traditional vascular risk factors and shown more severe WMH but less lacunes in neuroimaging, comparing to patients residing at low altitude. Hypobaric hypoxia at high altitude seems to have multiple effects on the occurrence and progression of CSVD, which represent as an aggravating factor in white matter impairment but a potential protective factor in lacunar infarction. Further studies are required to understand the prevalence of CSVD at different elevations and confirm the effect of chronic high altitude exposure on the incidence and outcome of CSVD.

## Data availability statement

The original contributions presented in the study are included in the article/supplementary material, further inquiries can be directed to the corresponding authors.

## Ethics statement

The studies involving human participants were reviewed and approved by the Ethics Committee of Peking University and the Ethics Committee of the Tibet Autonomous Region. The patients/participants provided their written informed consent to participate in this study.

## Author contributions

JS was involved in the data collection in Beijing, guided the neuroimaging evaluation, statistical analysis, interpreted the results, and drafted the manuscript. WF was responsible for data collection in Tibet and involved in the imaging evaluation, results interpretation, and important intellectual content. JZ participated in the data collection and imaging evaluation in Beijing. FL contributed to the data collection in Beijing. YHa, ZD, T, D, SJ, WZhao, and YHu contributed to the data collection in Tibet. WS, YHua, WZhang, and YZ were involved in design of the study, supervised the data collection process, the analysis and interpretation of the data, and revised the manuscript for intellectual content. All authors contributed to the article and approved the submitted version.
